# The efficacy and safety of neoadjuvant immunotherapy combined with chemotherapy for locally advanced gastric cancer: a single-center, real-world clinical study

**DOI:** 10.1007/s00262-025-04153-6

**Published:** 2025-09-18

**Authors:** Xuan Pan, Huayun Zhu, Xingwang Li, Siwen Liu, Shuchen Dong, Chao Yue, Pingping Wu, Yan Sun

**Affiliations:** 1 Department of Medical Oncology, Jiangsu Cancer Hospital & Jiangsu Institute of Cancer Research, The Affiliated Cancer Hospital of Nanjing Medical University, Nanjing, 210009 Jiangsu China; 2 Department of General Surgery, Jiangsu Cancer Hospital & Jiangsu Institute of Cancer Research, The Affiliated Cancer Hospital of Nanjing Medical University, Nanjing, 210009 Jiangsu China

**Keywords:** Gastric cancer, Neoadjuvant immunochemotherapy, PD-1 inhibitor, Pathological complete response, Safety

## Abstract

**Background:**

Immunotherapy has brought clinical benefits for patients with advanced or late-stage gastric cancer. This real-world study aimed to evaluate the efficacy and safety of neoadjuvant immunochemotherapy in patients with locally advanced gastric cancer (LAGC).

**Methods:**

Patients with LAGC (cT2-4bN1-3M0) were enrolled in this retrospective study. Patients received either neoadjuvant immunochemotherapy or chemotherapy alone before surgery. The primary endpoint was the pathological complete response (pCR) rate.

**Results:**

A total of 111 patients were included in this study, with 49 received neoadjuvant PD-1 inhibitors combined with chemotherapy (Group A) and 62 received chemotherapy alone (Group B). pCR rate of Group A was significantly higher than that of Group B (22.4% vs. 4.8%, *P* = 0.011). Compared with the clinical TNM stage before neoadjuvant therapy, 32 (65.3%) patients in Group A had T downstaging, 27 (55.1%) had N downstaging, and 42 (85.7%) had overall TNM downstaging. There was no significant difference in 1-year OS rate (100% vs. 93.5%; *P* = 0.195) and 1-year DFS rate (81.6% vs. 75.8%; *P* = 0.144) between the two groups. 11.7% patients in Group A and 9.8% patients in Group B experienced grade ≥ 3 adverse TRAEs, myelosuppression, elevated ALT/AST, nausea, and decreased appetite. However, there was no significant difference between the two groups (*P* = 0.240).

**Conclusions:**

Here, neoadjuvant immunochemotherapy suggested a potential clinical benefit of the pCR rate, T downstaging, and a favorable safety profile for LAGC. However, further multicenter, large-scale randomized clinical trials are urgently needed to confirm the long-term survival benefit.

**Supplementary Information:**

The online version contains supplementary material available at 10.1007/s00262-025-04153-6.

## Introduction

Globally, gastric cancer ranks fifth in incidence and fourth in mortality of malignant tumors [1]. Most gastric cancers are diagnosed in the advanced stage, and the 5-year survival rate for advanced gastric cancer remains poor [1]. Radical gastrectomy is the main treatment for locally advanced gastric cancer (LAGC) [2, 3]. Perioperative treatment of gastric cancer has been confirmed by a number of studies, which can reduce the tumor stage, increase the R0 resection rate, eliminate small metastases and improve overall survival, and does not increase postoperative complications and mortality compared with simple surgery [4–6]. Perioperative chemotherapy (FLOT regimen) has been shown to improve the median survival of LAGC to 50 months, with a 5-year overall survival (OS) rate of 45% [5]. Based on these findings, nowadays, perioperative chemotherapy has been widely recommended by multiple clinical guidelines.

Pathological response to neoadjuvant chemotherapy has been shown to predict survival in several neoadjuvant studies and be a prognostic factor of LAGC [7–9]. A retrospective analysis from an institutional database showed ypN + status substantially reduced survival for gastric or gastroesophageal junction (GEJ) adenocarcinoma patients with ypT0 status after neoadjuvant therapy [10]. Some patients with LAGC even achieved pathological complete response (pCR) after neoadjuvant chemotherapy. pCR refers to the fact that no significant tumor can be found in the resection specimens of gastric cancer after neoadjuvant therapy in general observation, and no tumor cells can be found under the microscope after extensive sampling in the primary tumor area. There is no standardized definition for pCR. Some clinical trials applied the pCR definition to the primary gastric tumor only [10, 11], whereas others have included the perigastric lymph nodes [12, 13]. It is well known that pCR, as the pathological basis for evaluating the efficacy of neoadjuvant therapy, is an important prognostic factor, and many large randomized controlled clinical studies have taken it as one of the main study endpoints [11], The 3-year disease-free survival (DFS) of pCR patients with gastric or GEJ adenocarcinoma was significantly higher compared with non-pCR patients receiving preoperative docetaxel-based chemotherapy [14].

The pCR rate varied greatly due to different neoadjuvant chemotherapy regimens, treatment modes, and treatment cycles. A retrospective study that collected data from the National Cancer Database (NCDB) analyzed the rate and predictor of pCR in gastric cancer patients who received neoadjuvant therapy. The pCR rate was only 2.2% but an independent predictor of survival [8]. In the large-scale prospective FLOT4-AIO study, the pCR rate of FLOT (fluorouracil + calcium folinate + oxaliplatin + docetaxel) as a perioperative therapy for patients with LAGC was 16%, and that of ECF (epirubicin + cisplatin + fluorouracil)/ECX (epirubicin + cisplatin + capecitabine) was only 6% [15]. The overall survival improved significantly in FLOT group compared with ECF/ECX group. In the RESOLVE study led by Professors Jiafu Ji and Shen Lin, the pCR rate of neoadjuvant chemotherapy using SOX (oxaliplatin+S−1) regimen for LAGC was 5.6% [4]. Perioperative chemotherapy with SOX showed a clinically significant improvement of 3-year DFS compared with adjuvant chemotherapy with capecitabine and oxaliplatin (CapeOx) [4]. F. Klevebro et.al found that neoadjuvant radio-chemotherapy resulted in higher pCR rate and higher R0 resection rate in patients with GEJ/esophageal adenocarcinoma [11].

Immune checkpoint inhibitors (ICIs), such as programmed cell death protein-1 (PD-1) inhibitors, can block the binding of PD-1 to its ligands, PD-L1 and PD-L2, reverse immune suppression [16, 17]. Growing evidence proves that PD-1 inhibitors have brought clinical benefits for patients with advanced or late-stage gastric cancer in first-line treatment [18–22]. Nivolumab was proved to be the first PD-1 inhibitor that improved OS along with progression-free survival (PFS) as first-line treatment for Her-2-negative advanced G/GEJ/esophageal adenocarcinoma in combination with chemotherapy versus chemotherapy alone [18]. Sintilimab + CapeOx regimen significantly improved OS for advanced gastric/GEJ adenocarcinoma [19]. The phase 3 KEYNOTE-811 study reported that pembrolizumab combined with first-line trastuzumab and chemotherapy (fluoropyrimidine and platinum-based therapy) improved PFS significantly for metastatic HER2-positive gastro-esophageal cancer [20]. With the advancement of ICIs from first-line treatment in late-stage disease to perioperative treatment in local advanced stage disease, more and more studies have found that neoadjuvant immunotherapy combined with chemotherapy can significantly increase the pCR rate in patients with LAGC. The KEYNOTE-585 trial reported that the pCR rate of pembrolizumab combined with FLOT chemotherapy in patients with LAGC was 12.9%, which was superior to chemotherapy alone [23]. In a national multicenter, prospective phase II randomized controlled study (PERSIST), the pCR rate of SOX chemotherapy in combination with sintilimab was 26.8%, significantly higher than that of control group (4.8%). Though the median event-free survival (EFS) and OS were not reached, the study will provide more effective guidance for the clinical practice of perioperative treatment [24]. Sintilimab + CapeOx improved the pCR rate in the neoadjuvant setting in locally advanced, resectable G/GEJ adenocarcinoma [25, 26]. The pCR and MPR rates of neoadjuvant sintilimab in combination with FLOT chemotherapy in patients with HER2-negative LAGC or GEJ adenocarcinoma were 17.2% and 55.2%. Better EFS, DFS, and OS were found in patients achieving pCR than non-pCR [27]. Lately, a multicenter, randomized phase Ⅲ trial (DRAGON IV/CAP 05) found that camrelizumab + low-dose rivoceranib and SOX improved pCR rate compared with SOX alone in patients with G/GEJ adenocarcinoma (18.3% vs. 5.0%) [28]. The results of the MATERHORN study were reported at the 2025 ASCO conference. Perioperative combination of durvalumab with FLOT regimen significantly improved EFS with a nearly 30% reduction in the risk of disease progression (NR vs. 32.8 m, HR = 0.71) and the pCR rate (19.2% vs. 7.2%) [29]. And the treatment was safe, well tolerated, and no new safety issues were identified. At present, the mode of neoadjuvant treatment, the types of ICIs, and chemotherapy regimen in neoadjuvant therapy differ greatly, and these are important factors affecting the pCR rate of neoadjuvant therapy.

Randomized controlled trials (RCTs) provide high-level evidence by comparing outcomes between groups under controlled conditions, ensuring causality assessment. Nevertheless, the strict inclusion criteria limit the generalizability of extrapolation in RCTs (such as KEYNOTE-585, MATERHORN, and DRAGON IV/CAP 05), resulting in the inability to represent the diversity of the real-world population. In RCTs, the treatment dosage, duration, and monitoring were strictly carried out in accordance with the protocol. However, there are variations in dosage adjustments, interruptions, or patient compliance in the real world. The real-world study (RWS) involves a broader range of populations (such as the elderly and patients with comorbidities), to verify the applicability of the therapy in “real patients.” The RWS also reveals the actual treatment mode (such as various PD-1 inhibitors, the number of immunotherapy cycles, reasons for dose adjustment) and its impact on the therapeutic effect. Therefore, the efficacy and safety of neoadjuvant immunotherapy combined with chemotherapy for LAGC need to be explored largely.

In our retrospective study, we evaluated the efficacy and safety of neoadjuvant PD-1 inhibitors combined with chemotherapy for LAGC, and found that the pCR rate was higher in patients with PD-1 inhibitors combined with chemotherapy compared with chemotherapy alone. Furthermore, there was no major safety concerns during the study.

## Methods

### Study design and patient cohort

This is a single-center, retrospective study conducted to compare the efficacy and safety of neoadjuvant PD-1 inhibitors combined with chemotherapy versus chemotherapy alone for LAGC.

**Fig. 1:**
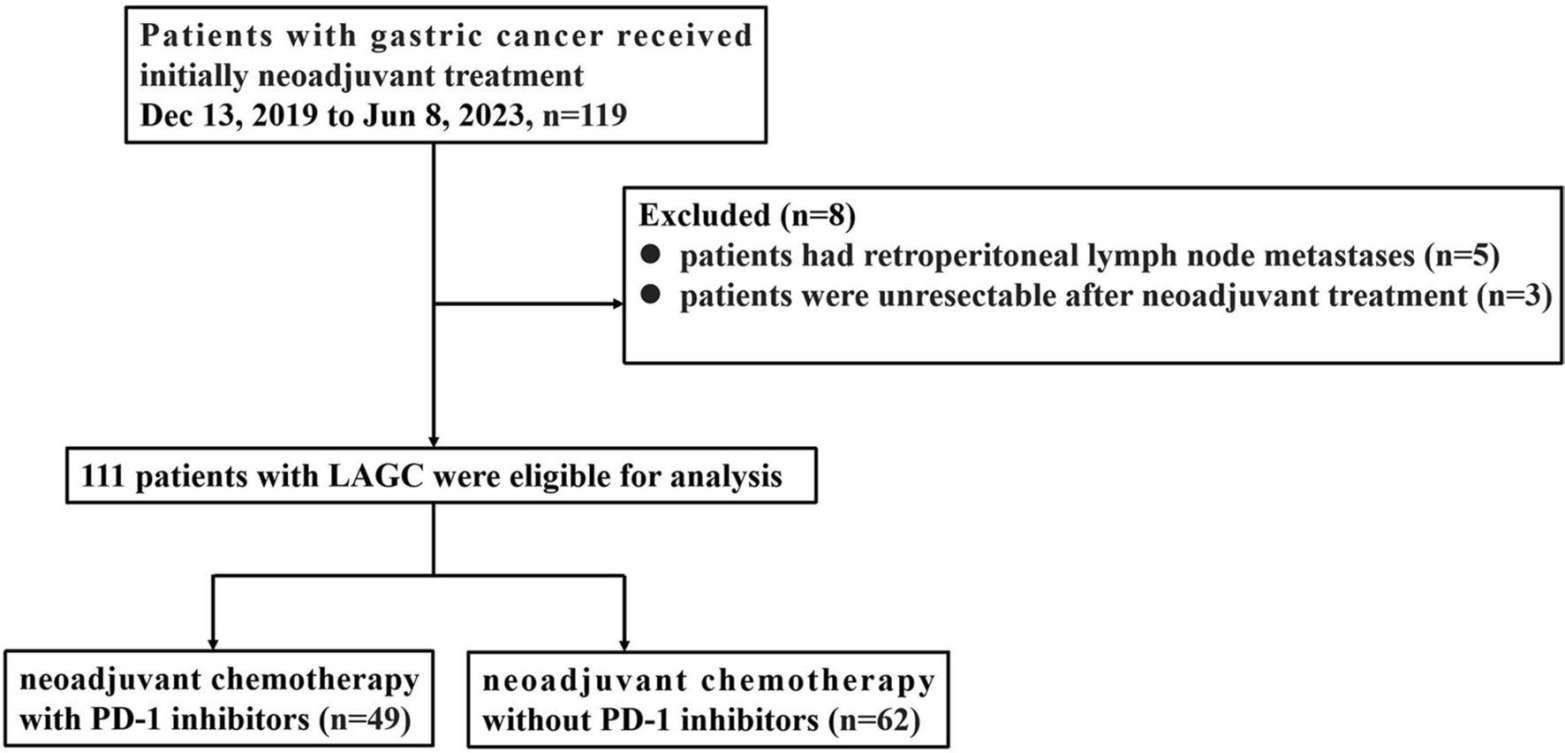
Flow diagram of study population

Clinical data were collected on 119 patients with LAGC as defined by T2 –4 primary lesion or any positive nodes (N_+_) without metastatic disease, receiving neoadjuvant therapy from December 13, 2019 to July 8, 2023 in Jiangsu Cancer Hospital. The main enrolled criteria: adult patients (over 18 years of age) who had not received prior treatment, histologically confirmed gastric/GEJ cancers at Jiangsu Cancer Hospital, Jiangsu, China, Eastern Cooperative Oncology Group performance status (ECOG PS) score 0–1, clinical stage was cT2-4bN1-3M0 detected by CT and/or MRI, based on American Joint Committee on Cancer (AJCC), 8th Edition guidelines, and at least one measurable lesion according to Response Evaluation Criteria in Solid Tumors (RECIST) v1.1, with adequate organ function to receive subsequent neoadjuvant therapy and surgery. Patients treated with PD-1 inhibitors combined with chemotherapy were included in Group A (*n* = 49), while those treated with chemotherapy without PD-1 inhibitors were included in Group B (*n* = 62). The process of patient selection is presented in Fig. 1.

### Procedure

#### Neoadjuvant therapy

Patients in Group A received chemotherapy + PD-1 inhibitors, while those in Group B received chemotherapy alone.

Anti-PD-1 monoclonal antibodies used in neoadjuvant therapy in this study included sintilimab (Innovent Biologics, Jiangsu, China), nivolumab (Bristol-Myers Squibb, New York, NY), tislelizumab (BeiGene, Beijing, China), toripalimab (Junshi Biosciences, Shanghai, China), and pembrolizumab (Merck, Rahway, NJ). Sintilimab, tislelizumab, and pembrolizumab were administrated 200 mg once every three weeks, toripalimab was administrated 240 mg once every three weeks, and nivolumab was administrated 240 mg once every two weeks.

There were three chemotherapy regimens included SOX, CapeOx, and nab paclitaxel + S-1. SOX consists of S-1 (50 mg, PO, bid, D1-14), and oxaliplatin (135 mg/m^2^, IV, D1). CapeOx consists of capecitabine (1000 mg/m^2^, PO, bid, D1-14), and oxaliplatin (135 mg/m^2^, IV, D1). Nab paclitaxel + S-1 consisted of nab paclitaxel (100–120 mg/m^2^, IV, D1, and 8) and S-1 (50 mg, PO, bid, D1-14). All patients received two to four cycles of neoadjuvant therapy before surgery.

The patients were reexamined with CT or MRI to evaluate the regression of the primary tumor and metastatic lymph nodes every six to eight weeks. A multidisciplinary team which consists of general surgeons, physicians, radiologists, and pathologists assessed the patients’ conditions, tumor downstaging, and patients’ willingness to operate.

#### Surgery

The objective of surgery was for R0 resection, referring to curative resection of gastric primary lesions and regional lymph nodes with no tumor cells at the margin of resection microscopically. Then, the surgeons assessed whether radical surgery could be performed and decided on the surgical protocol and decided on the surgical protocol according to guidelines, experience, and tumor board discussions. The surgical informed consent was signed before the operation. The surgical procedures included radical gastrectomy, proximal gastrectomy, and distal gastrectomy. Gastrojejunostomy was planned with Billroth II reconstruction, while esophagojejunostomy was planned with Roux-en-Y reconstruction, according to the extent of gastrectomy.

#### Assessments

The assessment of postoperative pathological type, ypTNM stage, and efficacy after neoadjuvant therapy were completed by two experienced pathologists. ypTNM stage was based on AJCC, 8th Edition guidelines. Ryan criteria of tumor regression grade (TRG) was used to evaluate the efficacy of neoadjuvant therapy, according to the amount of tumor residue and the degree of pathological regression. TRG 0, no tumor cells remained, including primary gastric tumor and lymph nodes (pathological stage ypT0N0cM0, means pCR). TRG 1, only single cell or small groups of cancer cells remain. TRG 2, tumor remains, but less than fibrotic interstitium. TRG 3 extensive residual tumor, no or few tumor cell necrosis.

PD-1 expression was evaluated using immunohistochemistry (IHC) on formalin-fixed, paraffin-embedded (FFPE) tumor samples with the PD-L1 IHC 22C3 pharmDx assay (Dako, Glostrup, Denmark). A combined positive score (CPS) was used to characterize the PD-L1 expression. CPS was calculated as: number of PD-1-positive cells (tumor cells, lymphocytes, macrophages) divided by number of all tumor cells × 100. PD-L1 positivity was defined as CPS ≥ 1.

The mismatch-repair (MMR) status was assessed by IHC for MLH1, MSH2, MSH6, and PMS2. The following monoclonal antibodies (Dako, Denmark) were used: MLH1 (clone: ES05, dilution 1:100), PMS2 (clone, EP51, dilution 1:100), MSH2 (clone, FE11, dilution 1:100), MSH6 (clone, EP49, dilution 1:100). Negative controls (without the primary antibody) and positive controls were included in each run to ensure the specificity and accuracy of the staining procedure.

Surgical safety and the incidence of surgery-related complications were assessed. During the treatment, all patients were monitored for toxicity. Adverse events of PD-1 monoclonal antibodies and chemotherapy were graded according to the NCI Common Terminology Criteria for Adverse Events (CTCAE) version 5.0.

#### Follow-up

After surgery, patient received postoperative treatment or clinical observation according to patients’ pathological reports. Follow-up was performed every three to six months for the first two years, and then every six to 12 months to five years. The key points of follow-up are physical examination, blood tests (including tumor markers, CEA, CA199), and chest, abdominal and pelvic enhanced CT imaging studies. DFS was defined as the time from surgery to recurrence. The last follow-up was performed on April 23, 2025.

### Statistics

All statistical analyses were performed using R software (version 4.3.3) and relevant packages. The Chi-square test was performed using the chisq.test function in the base stats package in R to examine the association between categorical variables. Expected frequencies were calculated for all categories to ensure the validity of the test. *P* value < 0.05 was considered statistically significant. Survival analysis was conducted to evaluate the impact of immunotherapy on patient outcomes, including OS and DFS. Kaplan–Meier survival curves were generated, and the log-rank test was applied to compare survival distributions between groups. Survival curves were visualized using ggplot2 (version 3.5.1) and survminer (version 0.4.9) packages in R.

Multivariate Cox proportional hazards regression analysis was performed to identify independent prognostic factors for OS and DFS. Similarly, multivariate binary logistic regression analysis was conducted to evaluate factors associated with pCR. All relevant clinical and pathological variables were included directly in the multivariate models without prior univariate screening. The results were presented as hazard ratios (HRs) or odds ratios (ORs) with 95% confidence intervals (CIs). All statistical analyses were conducted using SPSS software (version 27.0.1.0, IBM Corp., Armonk, NY, USA), and a two-sided *P* value < 0.05 was considered statistically significant.

## Results

### Patient characteristics and neoadjuvant treatment

Between December 13, 2019 to June 8, 2023, 119 patients with gastric cancer who received initially neoadjuvant treatment were screened. Five patients had retroperitoneal lymph node metastases (referred as M1), three patients were unresectable, and a total of 111 patients were included in this study. In the main cohort, 89 (80.2%) were male, and 22 (19.8%) were female. The median age was 65 (range, 35–79). Of the 111 patients, 102 (91.9%) had stage III disease with regional lymph nodes metastasis (N1-3). Furthermore, 50 (45%) of tumors were poorly differentiated, and 34 (30.6%) of them were moderately/poorly differentiated. Among them, 106 (95.5%) of them were gastric adenocarcinoma. Most adenocarcinomas are mixed ingredients, including mainly tubular adenocarcinoma, mucinous adenocarcinoma, low adhesion carcinoma (with signet-ring cell carcinoma), hepatoid adenocarcinoma, and so on. Patients of intestinal type, diffuse type, and mixed type were 36 (32.4%), 33 (29.7%), and 20 (18.0%), respectively. Fifty (45.0%) of the tumor were histologically graded G3, and 34 (30.6%) were G2–G3, suggesting that these tumors were poorly differentiated. Of 111 patients, 87 (78.4%) had PD-L1 CPS of one or more, and 88 (79.2%) were pMMR. Patient baseline characteristics were similarly well balanced in Group A and Group B. Detailed baseline clinicopathological characteristics of 111 patients are presented in Table 1.

**Table 1:** Baseline patient characteristics

Characteristics	Group A (n = 49)	Group B (n = 62)	*P* value
Age, median age	64 (35–78)	66 (39–79)	
Gender, n (%)			0.391
Male	37 (75.5)	52 (83.9)	
Female	12 (24.5)	10 (16.1)	
ECOG performance status, n (%)			
0	35 (71.4)	46 (74.2)	0.912
1	14 (28.6)	16 (25.8)	
Clinical T stage, n (%)			0.554
T2	1 (2.0)	4 (6.4)	
T3	17 (34.7)	16 (25.8)	
T4a	29 (59.2)	40 (64.5)	
T4b	2 (4.1)	2 (3.2)	
Clinical N stage, n (%)			0.804
N1	16 (32.7)	24 (38.7)	
N2	26 (53.1)	30 (48.4)	
N3	7 (14.3)	8 (12.9)	
Clinical TNM stage, n (%)			0.529
IIA	1 (2.0)	4 (6.5)	
III	46 (93.9)	56 (90.3)	
IVA (T4bNxM0)	2 (4.1)	2 (3.2)	
Histological grade, n (%)			0.874
G2	13 (26.5)	14 (22.6)	
G2–G3	15 (30.6)	19 (30.6)	
G3	21 (42.9)	29 (46.8)	
Lauren classification, n (%)			0.181
Intestinal type	16 (32.7)	20 (32.3)	
Diffuse type	12 (24.5)	21 (33.9)	
Mixed type	7 (14.3)	13 (20.9)	
Unknown	14 (28.6)	8 (12.9)	
Pathological type, n (%)			1.000
Adenocarcinoma	47 (95.9)	59 (95.2)	
Neuroendocrine carcinoma	2 (4.1)	3 (4.8)	
MMR status, n (%)			0.488
pMMR	39 (79.6)	49 (79.0)	
dMMR	1 (2.0)	4 (6.5)	
Unknown	9 (18.4)	9 (14.5)	
PD-L1 CPS %, n (%)			0.128
< 1	0 (0)	5 (8.1)	
≥ 1 and < 5	15 (30.6)	24 (38.7)	
> 5	24 (49.0)	24 (38.7)	
Unknown	10 (20.4)	9 (14.5)	

A total of 73 (65.8%) patients received SOX regimen for neoadjuvant chemotherapy. The patients who received CapeOx and nab paclitaxel + S-1 regimen were 24 (21.6%) and 14 (12.6%), respectively. In Group A, 29 (59.2%), 9 (18.4%), 6 (12.2%), and 3 (6.1%), 2 (4.1%) patients received sintilimab, tislelizumab, nivolumab, toripalimab, and pembrolizumab administration intravenously on the first day of therapy.

### Surgical treatment and complications

After neoadjuvant treatment, all patients in Group A and Group B underwent D2 radical gastrectomy successfully. The rate of R0 resection was 100%. In Group A, 34 (69.4%), 7 (14.3%), 8 (16.3%) of patients underwent total gastrectomy, distal gastrectomy, and proximal gastrectomy, respectively. While in Group B, 45 (72.6%), 7 (11.3%), 10 (16.1%) of patients underwent total gastrectomy, distal gastrectomy, and proximal gastrectomy, respectively.

In the entire cohort, surgical complications were observed in 14 patients in Group A, including pneumonia in nine patients, intraperitoneal infection in two patients, anastomotic leakage in two patient and gastroparesis in one patient. There were 10 patients with surgical complications in Group B, including pneumonia in seven patients, anastomotic hemorrhage in one patient, anastomotic leakage in one patient, and pleural effusion in one patient. All of these patients improved after symptomatic treatment. No patients underwent reoperation. No surgery-related deaths occurred in either group.

### Pathological response to neoadjuvant treatment

Pathological response was assessed in all patients. In Group A, 11 (22.4%) patients achieved pCR (Table 2). The absence of residual cancer cells in the primary site of surgical specimen was seen in two patients, but viable residual tumor cells were detected in their lymph nodes (one was ypT0N2, and one was ypT0N1). pCR rate of Group A was significantly higher than that of Group B (22.4% vs. 4.8%, *P* = 0.011).

**Table 2:** Pathological response after treatment

Tumor response	Group A (n = 49)	Group B (n = 62)	*P value*
pCR, n (%)	11 (22.4)	3 (4.8)	0.011
TRG			
1	10 (20.4)	7 (11.3)	
2	16 (32.7)	32 (51.6)	
3	12 (24.5)	20 (32.3)	
Pathological T stage, n (%)			0.003
ypT0	13 (26.5)	3 (4.8)	
ypT1a	1 (2.0)	4 (6.5)	
ypT1b	4 (8.2)	2 (3.2)	
ypT2	4 (8.2)	12 (19.4)	
ypT3	16 (32.7)	17 (27.4)	
ypT4a	11(22.4)	20 (32.3)	
ypT4b	0 (0)	4 (6.5)	
Pathological N stage, n (%)			0.858
ypN0	21 (42.9)	26 (41.8)	
ypN1	12 (24.5)	12 (19.4)	
ypN2	9 (18.4)	12 (19.4)	
ypN3	7 (14.2)	12 (19.4)	
Nerve infiltration, n (%)			0.483
Yes	18 (36.7)	28 (45.2)	
No	31 (63.3)	34 (54.8)	
Vascular tumor embolus, n (%)			0.718
Yes	18 (36.7)	26 (41.9)	
No	31 (63.3)	36 (58.1)	

Multivariate binary logistic regression analysis for pCR, adjusting for age, sex, chemotherapy regimen, surgical type, and combination of PD-1 inhibitors, revealed a significantly higher pCR rate in the PD-1 inhibitor group (OR: 6.154, 95% CI 1.503–25.190, *P* = 0.012) (Supplementary Table 1).

In Group A, seven (63.6%) patients achieved pCR with PD-L1 CPS of one or more. The PD-L1 CPS scores of other four patients were unknown (Supplementary Table 2). Pathological T stage after surgery in Group A was ypT0, ypT1a, ypT1b, ypT2, ypT3, and ypT4a in 13 (26.5%), 1 (2.0%), 4 (8.2%), 4 (8.2%), 16 (32.7%), and 11 (22.4%) patients, respectively, which were significantly different from those in Group B (*P* = 0.003). What’s more, in Group B, four patients were ypT4b. The rate of ypT3 + 4 was 55.1% and 66.1% in Group A and Group B, respectively. Pathological N stage after surgery in Group A was ypN0, ypN1, ypN2, and ypN3 in 21 (42.9%), 12 (24.5%), 9 (18.4%), and 7 (14.2%) patients, respectively (Table 2). Compared with the clinical TNM stage before neoadjuvant therapy, 32 (65.3%) patients in Group A had T downstaging, 27 (55.1%) had N downstaging, and 42 (85.7%) had overall TNM downstaging. While 36 (58.1%) patients in Group B had T downstaging, 35 (56.5%) had N downstaging, and 49 (79.0%) had overall TNM downstaging. The rate of positive nerve infiltration and vascular tumor embolus in Group A was lower than that of in Group B (36.7% vs. 45.2%, *P* = 0.483), (36.7% vs. 41.9%, *P* = 0.718). However, there was no statistical difference between two groups (Table 2).

### Survival outcome

**Fig. 2:**
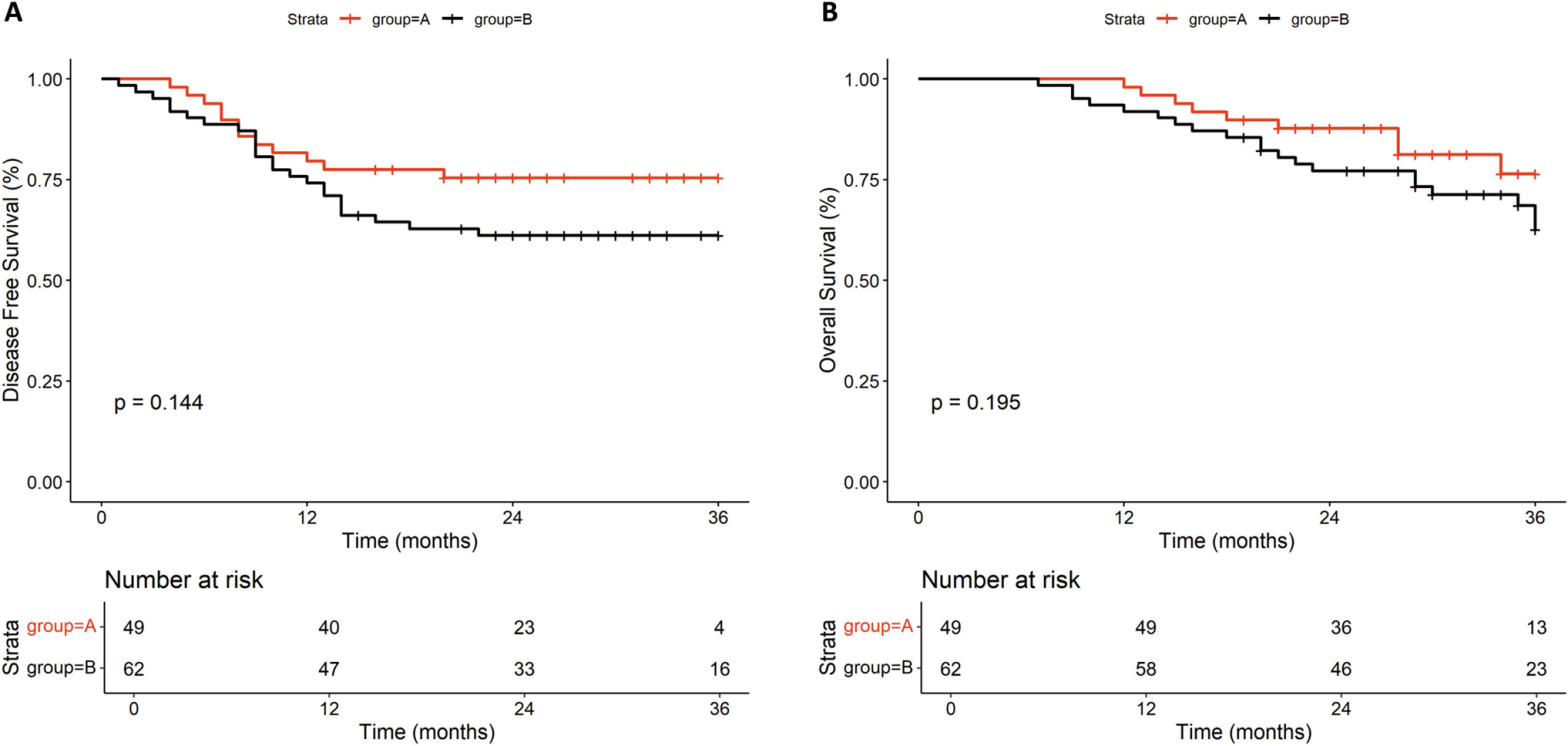
Kaplan–Meier estimates of DFS (**A**) and OS (**B**) of patients in Group A and Group B

All patients were followed up after surgery. As of data cutoff, the median follow-up was 30 months (range, 22–64 months). The median DFS and OS were not reached, and the 1-year DFS rate was 81.6% and 75.8% for Group A and Group B, respectively (*P* = 0.144). However, there was no significant difference in the 1-year DFS rate between two groups. The proportion of early recurrence in Group A was lower than that in the Group B (Fig. 2A). As shown in Fig. 2B, the 1-year OS rates of Group A were better than those of Group B (100% vs. 93.5%, *P* = 0.195). However, there was no statistical difference between two groups. Age, sex, combination of PD-1 inhibitor, chemotherapy regimen, and surgery type were all included in the multivariate Cox proportional hazards regression analysis for PFS and OS. The results showed no significant difference in PFS and OS in all these subgroups (Supplementary Table 3). Nevertheless, Group A showed a trend toward improved DFS and OS compared to Group B, though the difference did not reach statistical significance (data not shown).

A total of 24 patients developed recurrence and distant metastasis after surgery within one year. All of them were ypTNM stage II or III. Thirteen (54.2%) patients developed lymph node metastasis, including retroperitoneal lymph node, abdominal lymph node, mediastinal lymph node, and left supraclavicular lymph node metastasis. Seven (29.2%) patients developed hepatic metastasis. One patient underwent a colonoscopy due to pain and discomfort in the mid-abdomen. The endoscopist found that the splenic region of the colon was narrow and the endoscopic body could not pass. The pathology and immunohistochemistry of biopsy indicated that the tumor was a metastasis of gastric cancer. Subsequently, the patient underwent left hemicolectomy and mesenteric lymph node dissection. Intraoperative exploration revealed that the tumor was located at the upper margin of the left diaphragmatic horn, indicating that the tumor had recurred and invaded the splenic region of the colon. Combined with the morphology and immunohistochemistry of the tumor tissue from surgery, the diagnosis of metastasis of gastric cancer was made. Brain, ovarian, and bone metastases occurred in one, one, and two patients, respectively.

### Safety

In the main cohort, treatment-related adverse events (TRAEs) of any cause occurred in almost all the patients in Group A and 59 (95.2%) of patients in Group B during the neoadjuvant treatment period. As shown in Table 3, grade 3 or worse adverse events of any cause were reported in 11.7% patients in Group A and 9.8% patients in Group B, the most common TRAEs were myelosuppression, elevated ALT/AST, nausea, and decreased appetite. No grade 5 TRAEs and no treatment-related deaths occurred in either group.

**Table 3:** Neoadjuvant treatment-related adverse events

	Group A (n = 49)				Group B (n = 62)			
	Grade1-2	Grade3	Grade4	Grade5	Grade1-2	Grade3	Grade4	Grade5
Leucopenia	25 (51.0%)	3 (6.1%)	1(2.0%)	0	30 (48.4%)	3 (4.8%)	0	0
Neutropenia	22 (44.9%)	3 (6.1%)	1(2.0%)	0	25 (40.3%)	3 (4.8%)	0	0
Anemia	20 (40.8%)	2 (4.1%)	0	0	23 (37.1%)	2 (3.2%)	0	0
Thrombocytopenia	10 (20.4%)	1 (2.0%)	0	0	10 (16%)	1 (1.6%)	0	0
Nausea/vomiting	11 (22.4%)	2 (4.1%)	0	0	12 (19%)	2 (3.2%)	0	0
Elevated ALT/AST	12 (24.5%)	3 (6.1%)	0	0	14 (22.6%)	3 (4.8%)	0	0
Decreased appetite	18 (36.7%)	1 (2.0%)	0	0	19 (30.6%)	1 (1.6%)	0	0
Fatigue	8 (16.3%)	1 (2.0%)	0	0	9 (14.5%)	1 (1.6%)	0	0
Rash	10 (20.4%)	1 (2.0%)	0	0	3 (4.8%)	0	0	0
Pneumonia	4 (8.1%)	0	0	0	2 (3.2%)	0	0	0
Hyperthyroidism	4 (8.1%)	0	0	0	1 (1.6%)	0	0	0

## Discussion

The efficacy of immune checkpoint inhibitors combined with chemotherapy in the neoadjuvant therapy for LAGC varied greatly. In this retrospective study, we found that PD-1 inhibitors combined with chemotherapy improved pCR rate (22.4% vs. 4.8%) and the downstaging of T stage significantly compared to chemotherapy alone. All patients in underwent D2 radical gastrectomy successfully. The rate of R0 resection was 100%.

The clinical benefits of neoadjuvant chemotherapy in LAGC have been extensively studied. Preoperative chemotherapy can increase the radical resection rate and improve the prognosis. Nevertheless, the pCR rate reported before was not very satisfactory, more effective treatment is urgent needed. Pretreatment clinical stage, tumor size, pretreatment serum oncology indicators, neoadjuvant therapy mode, and the time interval between the end of neoadjuvant chemotherapy and surgery are all factors that affect post-neoadjuvant pCR rate for LAGC. Further, a large number of previous studies showed that the pCR rate varied greatly (2.2–26.8%) due to different treatment groups, different treatment modes, different treatment plans, and different cycles [4, 8, 15, 28]. Nowadays, the introduction of immunotherapy into perioperative treatment is expected to further reduce the recurrence rate and improve the cure rate. In our single-center RWS study, the pCR rate of PD-1 inhibitors combined with chemotherapy was 22.4%, the control group was 4.8%. In the KEYNOTE-585 trial (randomized phase 3 trial), the pCR rate of pembrolizumab combined with FLOT chemotherapy in patients with LAGC was 12.9%, while placebo combined with FLOT was only 2% [23]. The PD-1 inhibitors used in this study included sintilimab, nivolumab, tislelizumab, toripalimab, and pembrolizumab. The chemotherapy regimens included SOX, CapeOx, and nab paclitaxel + S-1. While in KEYNOTE-585 trial, the PD-1 inhibitor was pembrolizumab and the chemotherapy regimen was FLOT. Another multicenter real-world study found that PD-1 inhibitors combined with chemotherapy exhibited a higher pCR rate (14.4% vs. 6.4%), followed by laparoscopic gastrectomy and a lower proportion of early recurrence than chemotherapy alone among patients with LAGC [13]. Similar to our research, the PD-1 inhibitors and chemotherapy regimens were not of a single type. Furthermore, in the KEYNOTE-585 and other neoadjuvant RCTs for gastric cancer, the median age was typically around 60 years old. However, in real-world patients, the proportion of those aged 70 or above was even higher. So, RCTs (such as KEYNOTE-585, MATERHORN, and DRAGON IV/CAP 05) are well designed, with relatively fixed and idealized research plans, and the enrolled population is relatively limited. In the RWS, the patients included might be more extensive, and the treatment models are more individualized. Therefore, real-world studies can serve as a complement to RCT studies.

As mentioned above, PD-1 inhibitor combined with chemotherapy improved pCR rate of patients with LAGC compared with chemotherapy alone [13, 23, 30, 31]. However, the effect of neoadjuvant nivolumab monotherapy was limited in patients with resectable GC. The result of a phase I trial showed the pCR rate of nivolumab monotherapy was only 3.2% [32]. Thus, the synergistic effect of combined therapy warrants further investigation. In addition, new neoadjuvant therapy models have been explored. The pCR rate of neoadjuvant pembrolizumab-containing chemoradiation for GEJ adenocarcinoma was 22.6% [33]. Sintilimab in combination with chemoradiotherapy demonstrated promising pathological response and a favorable safety profile in the perioperative treatment for locally advanced G/GEJ cancers. The pCR rate increased to 38.2% [34].

Although pCR has good prognostic value in neoadjuvant therapy for LAGC, it can be used as a short-term indicator to predict the effect of neoadjuvant therapy [35]. However, the question of whether pCR can translate into survival benefits and replace long-term survival as a predictor of efficacy remains controversial. Hofheinz et al. showed that pCR was a predictive factor for the improvement of DFS in patients receiving neoadjuvant therapy combined with radical resection of gastric cancer, but not for the improvement of OS [36]. However, Ajani et al.’s findings showed that an increase in pCR rate did not ultimately translate into a survival benefit [37]. In addition, results of KEYNOTE-585 study showed that the pCR rate of immunotherapy combined with chemotherapy increased by 10% compared with chemotherapy alone, but the EFS and OS did not reach the prespecified primary endpoint. Therefore, the effect of pCR on long-term prognosis needs to be further evaluated. In the present study, 1-year DFS and 1-year OS of neoadjuvant immunochemotherapy were longer than that of chemotherapy alone. However, there was no statistical difference between two groups. This may be related to the relatively small sample size and short follow-up time.

As mentioned above, multiple studies have confirmed the exact efficacy of anti-PD-1 monoclonal antibody in neoadjuvant therapy, and immunochemotherapy can improve the pCR and MPR rates compared with chemotherapy alone. These anti-PD-1 monoclonal antibody mentioned in previous studies included pembrolizumab, sintilimab, toripalimab, and nivolumab. In this retrospective study, 49 patients received immunochemotherapy. 61.2% patients were treated with sintilimab, and the others were treated with tislelizumab, nivolumab, toripalimab, and pembrolizumab. RATIONALE-305 study showed that tislelizumab combined with chemotherapy compared with placebo combined chemotherapy as the first-line treatment significantly prolonged the survival of patients with locally advanced unresectable or metastatic G/GEJ adenocarcinoma when in, regardless of patients’ PD-L1 TAP score ≥ 5% or the whole population [38]. There are also many therapeutic options for neoadjuvant chemotherapy, such as CapeOx, SOX, FLOT, nab paclitaxel + S-1, and so on. In this study, the main research protocols are CapeOx, SOX, and nab paclitaxel + S-1. Nab paclitaxel has been proven effective as first- and second-line treatment of advanced gastric cancer [39–41]. GAPSO study demonstrated that nab paclitaxel + S-1 compared with oxaliplatin + S-1 improved the progression-free survival in advanced gastric cancer [41]. A phase 2 trial also showed the promising efficacy and good safety profile of sintilimab combined with concurrent chemoradiotherapy (S-1 and nab paclitaxel) in locally advanced G/GEJ adenocarcinoma [34]. A prospective Dragon-VII trial was conducted to evaluate the safety and efficacy of nab paclitaxel, S-1 and sintilimab as adjuvant therapy for patients with stage IIIC gastric cancer [42].

In our study, the toxicity of neoadjuvant immunochemotherapy was manageable and the most common TRAEs were myelosuppression (anemia, leukopenia, and neutropenia), consistent with previous findings [13]. Others were decreased appetite, elevated ALT/AST and nausea/vomiting. Most TRAEs were grade 1 or grade 2. The incidence of grade 3–4 adverse events in Group A and Group B was 11.7% and 9.8%, respectively, with no significant difference (*P* = 0.24). One patient experienced Grade 3 rash, probably related to anti-PD-1 antibodies. Immunotherapy was suspended in this patient. After topical and oral glucocorticoid therapy, the rash was reduced to grade 1, then immunotherapy continued. Except for rash, other common immune-related adverse events (irAEs) were pneumonia and hyperthyroidism. Thus, the toxicity of the addition of a PD-1 inhibitor to chemotherapy during neoadjuvant period was manageable compared to chemotherapy alone. Nevertheless, with the increasing application of immunotherapy, irAEs deserve our close attention.

A national multicenter retrospective study of locally advanced gastric cancer in the Chinese population found that patients with earlier stage, better differentiation, and chemotherapy combined with immunotherapy were more likely to achieve pCR [43]. PD-L1 CPS score was proven to predict the efficacy of anti-PD-1 therapy. Patients with high expression of PD-L1 (CPS ≥ 10) in the TME had a significantly higher pCR rate than those with low expression received neoadjuvant pembrolizumab-containing chemoradiation [33]. Wei et.al found that patients with PD-L1 CPS ≥ 5 achieved higher pCR rate than these with PD-L1 CPS < 5. In addition, pCR rate was associated with CD3 + T cells, CD56 + NK cells, and the M1/M1 + M2-like macrophage infiltration at baseline [34]. A retrospective study showed that the pCR and MPR rates of neoadjuvant immunotherapy were significantly higher in patients with PD-L1 CPS ≥ 5 [31]. In our study, we did not see such a significant difference. However, 66.7% of patients in Group A achieved pCR with PD-L1 CPS of one or more. The possible reason was the small sample size, and PD-L1 CPS of some patients were unknown in Group A. Mismatch-repair (MMR) status is also a biomarker of the efficacy of PD-1 inhibitors. A phase 2 study showed that MMR status predicted clinical benefit from pembrolizumab in patients with colorectal cancer, stomach cancer, and so on. Patients with deficient MMR (dMMR) were more responsive to PD-1 blockade than those with proficient mismatch repair (pMMR) [44]. The dense immune infiltration and Th1-associated cytokine-rich environment were observed in dMMR tumors [45, 46]. The pCR rate in patients with dMMR was higher than that in those with pMMR, suggesting that these patients may benefit from chemotherapy combined with PD-1 inhibitor [30]. In the present study, only one patient in Group A was dMMR status but not achieved pCR. Given the established association between dMMR and enhanced response to immunotherapy, the limited presence of dMMR tumors in our cohort may have influenced overall treatment outcomes. Further studies in larger, biomarker-selected populations are warranted to explore the impact of MMR status in this therapeutic context.

The real-world study still had some limitations. Firstly, this was a single-center study without a randomized control group. Thus, our results may be influenced by local treatment protocols and patient selection biases that limit generalizability to broader populations. Secondly, due to the small sample size, the effects of different PD-1 inhibitors on pCR and survival were not compared. Thirdly, our follow-up time was short, and the DFS and OS data were not mature. The 1-year DFS and 1-year OS in immunochemotherapy group were better than the chemotherapy alone group, but there was no statistical difference between the two groups. After that, we will continue to follow up these 111 patients. Lastly, the number of patients was small, our study may have been underpowered to detect statistically significant differences in some clinical outcomes.

In our real-world study, PD-1 inhibitor combined with chemotherapy suggests a potential clinical benefit of the pCR rate, T downstaging, and a favorable safety profile in the neoadjuvant therapy for LAGC. Further, multicenter, large-scale randomized clinical trials are warranted to confirm the long-term survival benefit.

## Supplementary Information

Below is the link to the electronic supplementary material.

Supplementary file1 (DOCX 15 kb)

Supplementary file2 (DOCX 14 kb)

Supplementary file3 (DOCX 15 kb)

## Data Availability

If someone wants to request the data, or have queries from this study, please contact Dr. Xuan Pan (panxuan0214@163.com).

## Abbreviations

CPS: Combined positive score
DFS: Disease-free survival
EFS: Event-free survival
G/GEJ: Gastric/gastroesophageal junction adenocarcinoma
irAEs: Immune-related adverse events
ICIs: Immune checkpoint inhibitors
LAGC: Locally advanced gastric cancer
MPR: Major pathological response
dMMR: Mismatch-repair deficiency
MMR: Mismatch repair
OS: Overall survival
pCR: Pathological complete response
pMMR: Proficient mismatch repair
PD-1: Programmed cell death protein-1
PFS: Progression-free survival
RCTs: Randomized controlled trial
RWS: Real-world study
TRAE: Treatment-related adverse events
TRG: Tumor regression grade
