# Supplementary material for: The efficacy and safety of neoadjuvant immunotherapy combined with chemotherapy for locally advanced gastric cancer: a single-center, real-world clinical study
Source: Cancer Immunol Immunother. 2025 Sep 18;74(10):311. doi: 10.1007/s00262-025-04153-6 (PMC12446125; doi:10.1007/s00262-025-04153-6)
Supplement: Supplementary file 1 — Supplementary file1 (DOCX 15 kb) [file 262_2025_4153_MOESM1_ESM.docx]

**Supplementary Table 1. Multivariate binary logistic regression analysis identifying predictors of pCR.**

|  | **pCR** | | |
| --- | --- | --- | --- |
|  | **OR*** | **95%CI** | ***P* value** |
| **Sex** | 0.627 | 0.155-2.546 | 0.514 |
| **Age** | 0.424 | 0.121-1.481 | 0.179 |
| **PD-1 Antibody** | 6.154 | 1.503-25.190 | 0.012 |
| **Chemotherapy Regimen (CR)** | | | |
| **CR1** | [Reference] | | |
| **CR2** | 0.69 | 0.177-2.696 | 0.594 |
| **CR3** | 0.225 | 0.021-2.378 | 0.215 |
| **Surgery Type (ST)** | | | |
| **ST1** | [Reference] | | |
| **ST2** | 7.726 | 0.589-101.360 | 0.119 |
| **ST3** | 3.170 | 0.312-32.191 | 0.329 |

*OR: odds ratio. CR1 CapeOX, CR2 SOX,CR3 nab paclitaxel+S-1. ST1 distal gastrectomy, ST2 proximal gastrectomy, ST3 radical gastrectomy.
