# Supplementary material for: The efficacy and safety of neoadjuvant immunotherapy combined with chemotherapy for locally advanced gastric cancer: a single-center, real-world clinical study
Source: Cancer Immunol Immunother. 2025 Sep 18;74(10):311. doi: 10.1007/s00262-025-04153-6 (PMC12446125; doi:10.1007/s00262-025-04153-6)
Supplement: Supplementary file 2 — Supplementary file2 (DOCX 14 kb) [file 262_2025_4153_MOESM2_ESM.docx]

**Supplementary Table 2. The association of PD-L1 CPS score and pathologic response in Group A, which was evaluated using the chi-square test (*P* =0.284).**

| TRG | CPS < 1 | CPS ≥ 1, < 5 (n = 15) | CPS ≥ 5 (n = 24) | CPS unkown |
| --- | --- | --- | --- | --- |
| 0 | 0 | 2 | 5 | 4 |
| 1 | 0 | 2 | 5 | 3 |
| 2 | 0 | 3 | 10 | 3 |
| 3 | 0 | 7 | 4 | 1 |
