# Supplementary material for: The efficacy and safety of neoadjuvant immunotherapy combined with chemotherapy for locally advanced gastric cancer: a single-center, real-world clinical study
Source: Cancer Immunol Immunother. 2025 Sep 18;74(10):311. doi: 10.1007/s00262-025-04153-6 (PMC12446125; doi:10.1007/s00262-025-04153-6)
Supplement: Supplementary file 3 — Supplementary file3 (DOCX 15 kb) [file 262_2025_4153_MOESM3_ESM.docx]

**Supplementary Table 3. Multivariate cox regression analysis of PFS and OS**

|  | **DFS** | | | **OS** | | |
| --- | --- | --- | --- | --- | --- | --- |
|  | **HR*** | **95%CI** | ***P* value** | **HR** | **95%CI** | ***P* value** |
| **Sex** | 0.579 | 0.276-1.214 | 0.148 | 0.712 | 0.303-1.672 | 0.436 |
| **Age** | 0.865 | 0.433-1.727 | 0.680 | 0.740 | 0.343-1.597 | 0.443 |
| **PD-1 Antibody** | 0.622 | 0.303-1.276 | 0.195 | 0.683 | 0.299-1.563 | 0.367 |
| **Chemotherapy Regimen (CR)** | | | | | | |
| **CR1** | [Reference] | | | [Reference] | | |
| **CR2** | 1.269 | 0.508-3.169 | 0.610 | 2.653 | 0.770-9.141 | 0.122 |
| **CR3** | 0.674 | 0.165-2.756 | 0.583 | 1.560 | 0.309-7.874 | 0.590 |
| **Surgery Type (ST)** | | | | | | |
| **ST1** | [Reference] | | | [Reference] | | |
| **ST2** | 0.833 | 0.200-3.464 | 0.801 | 1.174 | 0.253-5.454 | 0.838 |
| **ST3** | 1.276 | 0.430-3.782 | 0.661 | 1.399 | 0.401-4.883 | 0.598 |

*HR: hazard ratio. CR1 CapeOX, CR2 SOX,CR3 nab paclitaxel+S-1. ST1 distal gastrectomy, ST2 proximal gastrectomy, ST3 radical gastrectomy.
